# The Science of Growth Monitoring: Beyond the Basics

**DOI:** 10.3390/children13020162

**Published:** 2026-01-23

**Authors:** Melodee Liegl, Amy Y. Pan

**Affiliations:** 1 Department of Pediatrics, Medical College of Wisconsin, 8701 Watertown Plank Road, Milwaukee, WI 53226, USA; 2 Center for Microbiome Research, Medical College of Wisconsin, 8701 Watertown Plank Road, Milwaukee, WI 53226, USA

**Keywords:** growth charts, CDC, WHO, percentile, Z-score

## Abstract

Growth charts are widely used as a clinical and research tool to assess physical growth performance of infants, children, and adolescents. They have been widely accepted as indicators of health and wellness. CDC and WHO growth charts are well known and used for tracking childhood growth. The differences between WHO and CDC growth curves are largely attributable to distinct reference population and curve construction methodologies. The aim of this review is to focus on the construction, utilization, as well as clinical significance of the CDC and WHO growth charts.

## 1. Introduction

It is essential for health-care providers to routinely check children’s pattern of growth. Serial measurements in children are recommended to help identify abnormal patterns of weight gain and physical growth which, subsequently, may be associated with morbidity and mortality. Therefore, growth charts have been used to monitor growth in individual children to support early identification and intervention for those with growth disturbances, such as childhood obesity and poor growth. Researchers, on the other hand, use growth charts to describe the growth status in a group of children and investigate the relationship with health-related predictors and outcomes.

The US Center for Disease Control and Prevention (CDC) 2000 growth charts [1] are considered reference because it describes the growth of children in the United States during 1963–94 and the growth of an individual child or a group of children and how they can compare with that of the reference population. Whereas the World Health Organization (WHO) growth charts are an international growth standard for children aged 0–59 months [2] that describe how healthy children should grow under optimal environmental and health conditions. In addition to CDC and WHO growth charts, alternatives have been developed to accommodate specific conditions such as prematurity or chronic illnesses. The aim of this review is to focus on the construction, utilization, as well as clinical significance of the CDC and WHO growth charts.

## 2. CDC and WHO Growth Charts

### 2.1. Reference Population

A percentile indicates what percent of the reference population is below that particular value. Underweight and obesity are defined as less than 5th and above 95th percentiles, respectively. However, since 1976, the prevalence of underweight among children and adolescent ages 2–19 years has been less than 5% with an estimated 4.1% in 2017–18 in the US according to CDC [3]. Conversely, in recent years, obesity rates have been growing rapidly. Approximately 19.3% of children and adolescents aged 2–19 years have obesity in the years of 2017–18 [4]. Clearly, the definition of underweight or obese is outdated. The CDC growth charts are revised from the National Center for Health Statistics (NCHS) growth charts. The NCHS growth charts used national survey data from the National Health Examination Survey (NHES) II (1963–65), NHES III (1966–70), and the National Health and Nutrition Examination Survey (NHANES) I (1971–74) as well as the longitudinal growth study of the Fels Research Institute in Yellow Springs, Ohio (1929–75). Additional national survey data such as NHANES II (1976–80), NHANES III (1988–94), and other supplemental data sets were added to the original data for the development of the CDC growth charts. It is worth noting that all the data were collected prior to 1994.

CDC growth reference is based on older data from a single country, in contrast, WHO growth charts employed data primarily collected through the Multicentre Growth Reference Study (MGRS) [5], a study conducted in 1997–2003 in six cities around the world: Davis, California, USA; Muscat, Oman; Oslo, Norway; and Pelotas, Brazil; and in selected affluent neighborhoods of Accra, Ghana, and South Delhi, India. The sample population used to generate the WHO growth chart for children aged < 2 years is from the longitudinal component of the MGRS study, where 882 distinct children were measured at different follow up points (18, 973 observations). The MGRS had strict inclusion criteria: no known environmental constraints on growth; adherence to MGRS feeding recommendations; no maternal smoking before or after delivery; single term birth; and no significant morbidity [5]. The feeding criteria were exclusively/predominantly breastfeeding for at least 4 months, complementary foods by 6 months of age, and partially breastfeeding until at least age 12 months. Therefore, all the sample population for the WHO were breastfed for 12 months while only 50% of the sample population for the CDC were ever breastfed. In addition, only very low birth weight (<1500 g) infants were excluded for the CDC reference population. Also, children who lived with chronic disease or in sub-standard living conditions were not excluded for CDC charts. Furthermore, weight and length data from birth to 2 months of age were not available in national survey data and the birth lengths were obtained exclusively from Wisconsin State and Missouri State for the CDC charts.

### 2.2. Construction of Growth Charts

Cole and Green [6] developed the LMS method which provided a way to account with skewness data commonly found in the distribution of height, weight, and other anthropometry measurements. This method also allowed for the development of smoothed curves and calculation of Z-scores simultaneously. The LMS parameters are the power for Box-Cox transformations to normality (L), the median (M), and the coefficient of variation (S). Based on the assumption that the residuals follow a normal distribution, these parameters can be generated from the empirical data using the maximum penalized likelihood. The L, M, S parameters are then used to construct the percentiles.

CDC employed a two-stage approach to generate the growth curve. Statistical parametric and nonparametric smoothing techniques were applied to the observed data to produce the smoothed curves for selected percentiles (e.g., 3rd, 5th, 10th, 25th, 50th, 75th, 90th, 95th, and 97th). For each specific age, a modified LMS method was used where best solutions were generated for the three parameters of L, M, and S simultaneously by solving a system of equations. This way, the final curves obtained are similar to the smoothed percentile based on the data and continuous Z-scores can then be acquired.

WHO excluded children who had extreme values to prevent the effects of unhealthy children before the construction of the growth curves [2]. Box-Cox power exponential (BCPE) distribution was selected out of five distributions: where µ—median, σ—coefficient of variation, ν—power of the Box-Cox transformation, and τ—power of exponential parameter (related to the kurtosis). Cubic spline was employed for curve smoothing. Rigby and Stasinopoulos [7] developed generalized additive models for location, scale and shape (GAMLSS) which is an extension of the LMS method. This allows modeling the mean/median of the growth variable with a variety of distribution (normal, skewed, and/or kurtotic) as well as other parameters. In practice, WHO curves omitted any kurtosis adjustment, therefore, it was equivalent to models fitted by the LMS method.

In the construction of the CDC growth curve, data below 3rd percentile or above 97th percentile were not used for the LMS parameter calculations. Flegal et al. [8] employed the CDC-supplied LMS parameters for extrapolation for the 99th percentile and the fit to the empirical data was poor. Conversely, WHO excluded the observations outside the ±3 standard deviation (SD) (or ±2 SD for the cross-sectional sample) of the sample median prior to the construction of the growth curve. However, this adaption may have trimmed the true extreme observations which were not caused by measurement errors. Therefore, the WHO estimated 99th percentile might not be the actual 99th percentile. Nonetheless, caution needs to be exercised when applying WHO or CDC growth chats to extremely high or low percentiles.

### 2.3. Differences Between CDC and WHO Growth Charts

The differences between WHO and CDC growth curves are largely attributable to distinct reference populations and curve construction methodologies (Table 1). de Onis et al. [9] reported the major difference was the weight-for-age curve during infancy where the WHO charts showed a rapid weight gain compared to the CDC charts. The CDC charts reflect a shorter and heavier sample than the WHO sample. While the application of WHO growth chart produces lower prevalence of undernutrition after the first 6 months and higher prevalence of overweight and obesity. Breastfeeding in the WHO samples is likely the primary cause of this difference. Instead of using the empirical data, the CDC infant curves were modeled using a linear model before calculating the L, M, S parameters due to the lack of data from birth to 2 months, which may imprecisely represent the actual growth pattern during this period. For infants 0–2 years of age, a direct comparison of the CDC and WHO charts [10] stated the use of WHO charts, more US infants would be above the 95th percentile, fewer US infants would be below the 5th percentile for weight-for-age, and more US infants would be below the 5th percentile using length-for-age. In addition to the difference of the reference populations, small sample size when stratified per gender and age group for CDC charts may also play an important role.

### 2.4. Recommendations

**Figure 1:**
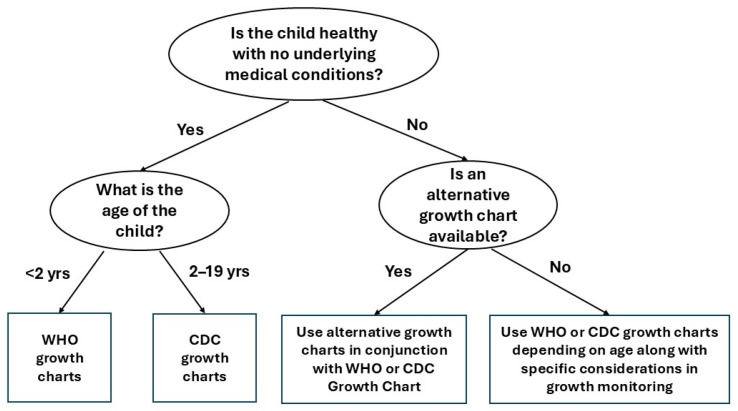
A decision tree for growth chart selection.

CDC, the National Institutes of Health (NIH), and the American Academy of Pediatrics (AAP) convened a meeting in June, 2006 where experts recommended the 2006 WHO international growth standard should be used to assess the growth among all children < 24 months and 2.3rd and 97.7th percentiles or values of 2 SDs above or below the median should be used to identify children with potential growth problems [11]. For children older than 2 years of age, the continued use of CDC growth charts was recommended (Figure 1).

This recommendation may cause some problems in transition, for example, discrepancy between classification by CDC and WHO for the same child at 24 months creates a disjunction. However, at 24 months, the transition between supine length and height measurement has already contributed to the disjointedness.

## 3. Body Mass Index and Percent Body Fat

Over the past decades, the prevalence of childhood overweight and obesity has escalated dramatically, leading to a significant public health crisis globally. As a result, this lead to increased morbidity and premature mortality in adulthood [12,13]. Body mass index (BMI), defined as weight (kg)/height^2^ (m), for age growth chart has been widely used to assess overweight and obesity. Appropriate cut-off points are essential for health providers to use BMI as a valid assessment tool. According to the CDC reference data, the expert committee recommended a BMI of 85th–94th percentile is overweight and ≥95th percentile is obesity [14]. WHO growth charts have consistently higher cut-off points than the CDC growth charts if using ≥95th percentile [15]. WHO Z-scores show a higher classification of overweight and obesity and a lower classification of underweight in different pediatric populations worldwide compare to CDC Z-scores [15,16,17,18,19,20].

The 2000 CDC BMI-for-Age growth charts do not show percentile curves above the 95th percentile and it is not recommended for use in children with extremely high BMI values, i.e., above the 97th percentile. In 2022, the CDC released the Extended BMI-for-Age growth charts based on additional data from 1999–2016 and updated statistical methods for children and adolescents with high BMI values [21,22], which provided detailed information beyond the 95th percentile by including 4 additional growth curves (the 98th, 99th, 99.9th, and 99.99th percentiles). The AAP guidelines for the treatment of obesity recommends using percentages of the 95th BMI-for-age percentile to indicate different levels of severe obesity. Childhood obesity is defined as having a BMI ≥ 95th percentile while severe obesity is defined as BMI ≥ 120% above the 95th percentile for age and sex, which approximates the 99th percentile [23].

Freedman and Sherry reports the Pediatric Rosetta Study, roughly 75% of children with a BMI at ≥95th CDC percentile had excess body fatness [24]. However, only 20% of children with excess body fatness had a BMI ≥ 99th CDC percentile. They concluded the BMI at ≥95th CDC percentile is a moderately sensitive and a specific indicator of excess fatness in children. In addition, Harrington et al. suggested the 95th CDC BMI percentile is a sensible and useful threshold with regards to predicting high visceral adipose tissue, total body fat mass, and cardio metabolic risk in children and adolescents [25].

The Bogalusa Heart Study examined over 2000 school children between 2 to 17 years of age and reexamined these children at ages 18 to 37 years to investigate the association between childhood BMI and adult levels of adverse risk factors such as lipids, insulin, and blood pressure [26]. The results indicated 77% of the children who had a BMI ≥ 95th percentile had a BMI of ≥30 kg/m^2^ as an adult but only a small percentage (7%) of normal weight children became obese later. Moreover, childhood overweight was correlated with adulthood cardiovascular risk factors.

It is the excess adipose tissue instead of the excess weight that causes the comorbid conditions. Adipose tissue has been viewed as a secretory organ that produces a wide range of bioactive substances, such as leptin and adiponectin, which may affect the function and structural integrity of the cardiovascular system [27]. The fact that BMI fails to distinguish between increased mass in the form of fat, lean tissue or bone results in surrogate measures gives misleading information [28]. Although BMI cannot distinguish between fat and fat-free mass, it is the only practical measure to use in clinical settings for this purpose [29,30]. Body fat measured by dual-energy radiograph absorptiometry (DEXA), 4-compartment model (combining measurements of total body water, body density, and total body bone mineral to estimate a fourth component—body fat), bod pod (air displacement plethysmography) or bioelectrical impedance are considered superior indicators of obesity.

Studies have shown that the relationship between BMI and body fatness may depend on age and sex, and may be different across racial or ethnic groups [31,32]. A cross-sectional study of 192 healthy children aged 7 to 17 years found body fat percentages were lower in more sexually mature adolescents compared with less sexually mature children of similar BMI [33]. Moreover, the study indicated that after controlling for BMI and maturation stages, boys have a lower body fat percentage than girls and White subjects have a higher body fat percentage than Blacks. As body composition and fat distribution vary across different racial and ethnic groups, obesity may be overestimated in Black populations and underestimated in Asian and Hispanic populations using BMI [34,35]. Consequently, this can lead to misdiagnosis or mismanagement of obesity across different ethnic groups.

It has been suggested that different BMI cut points should be set for overweight and obesity for Asians and Blacks [36,37]. Freedman et al. [38] examined DEXA estimated percent body fat among 1104 healthy 5–18 years old children and suggested equivalent levels of BMI-for-age; Black children had approximately 3% less body fatness than White children and Asian girls had 1% higher levels of body fat percentage than White girls. Additionally, these racial/ethnicity differences varied at different levels of BMI-for-age, i.e., higher percentage of body fat in Asian girls and lower percentage of body fat in Black boys were more evident among relatively thin children.

McCarthy et al. [39] constructed body fat reference curves using bio-impedance measured body fat in 1985 Caucasian schools and colleges located in Southern England for children aged 5–18 years of age. This direct measure of adiposity may have helped reduce the misclassification in overweight or obesity. However, the reference population is Caucasian and percent body fat exhibits significant heterogeneity across different ethnic populations. The limitations of body fat reference curves indicate they may not be appropriate for all children. When utilizing BMI cut points as an estimate of percent body fat, it is crucial to consider gender, race, maturity stage, and the distribution of adipose tissue. Moreover, the CDC growth charts are not accurate beyond the 97th percentile because the data above that were not used and extrapolation was not advised.

Given the limitations of BMI and discrepancies between BMI and percent body fat, the Lancet Diabetes & Endocrinology Commission provided objective criteria to diagnose obesity [40]. The Commission recommended body composition or at least one anthropometric measurement such as waist circumference, waist-to-hip-ratio, or waist-to-height ratio should be used to confirm excess adiposity in addition to BMI. However, this is yet to be validated in the pediatric population.

## 4. Alternative Growth Charts

Overall, growth charts are used for the general pediatric population. However, children with Eponymic Syndromes such as Down syndrome, Turner’s syndrome, Prader-Willi’s syndrome, or other specific conditions (premature infants, cerebral palsy, or fragile X syndrome) may have significantly different growth patterns than normal children. The reference populations are not representative of the patterns of growth for children with special conditions, therefore, alternative growth charts should be used in conjunction with WHO or CDC growth charts to correctly monitor their growth and also use as a comparison (Figure 1).

### 4.1. Premature Infants

Premature infants refer to babies that are born before 37 completed weeks of gestation and are at a higher risk for complications, including impairments in growth development [41]. It is crucial to perform medical and nutritional assessments for these infants and monitor their growth closely. Growth charts and intrauterine growth curves allow parents and health professionals to check children’s growth statuses and evaluate infants before term, respectively.

In 1970s, Babson and Benda [42] developed growth graphs to assess infants with various gestational ages (GAs). Limitations of the study include small sample size, older data, start at 26 weeks of gestation, and 500-g increments. Fenton then conducted a meta-analysis and updated the chart with recent data and a new format for use in neonatal intensive care units (NICU) [43]. As early as 22 weeks gestational age to 10 weeks post-term, the updated charts can be used for infants to compare individual infant’s growth with the fetus as well as the term infant. The INTERGROWTH-21st growth standards were developed from a large, multicenter study conducted in eight countries (Brazil, China, India, Italy, Kenya, Oman, UK, and the USA) [44]. It can be used across different settings as it describes the optimal growth for preterm infants regardless of their ethnicity or location. Comparison of Fenton and INTERGROWTH-21st growth standards of premature babies in different samples showed controversial results and differences, indicating the need for harmonization in growth metrics [45,46].

Olsen et al. [47] created a new set of growth curves based on contemporary, large, racially diverse singleton infants born in the US (1998–2006) with a final sample size of >250,000. The new charts include weight-, length- and head circumference-for-GA, which are also stratified by sex. The new curves were compared with the Lubchenco curves [48], which were based on full-term or premature infants admitted to the Colorado General Hospital from 1948–61 and were commonly used in the NICU. At younger GAs, the new curves had lower average weights, lengths, and head circumferences compared to the Lubchenco and vice versa for the older GAs [47]. They also suggested that small-for-GA (SGA) and large-for-GA (LGA) infant’s categorization based on the Lubchenco curve were not accurate. Taken together, these new curves offered an updated tool to the health professionals to evaluate a preterm infants’ growth status in NICUs.

The infant stage is a crucial period of development where many conditions can cause permanent changes on the brain and health. However, it is difficult to recognize infants with health problems at the earlier gestational age. Infants’ weight, length, and head circumference measurements plotted in the intrauterine growth charts will add important information to early diagnosis or identify high risk infants with various medical conditions.

### 4.2. Growth Charts for Cerebral Palsy

Children with cerebral palsy are known to grow poorly. Since the first growth reference standard for children with quadriplegic cerebral palsy (QCP) provided by Krick et al. [49], more growth charts are published [50,51] based on this sub population of children. The most recent growth charts constructed by Brooks et al. are based on the study in over 25,000 children with cerebral palsy [52]. They investigated the association between weight-forage percentile, the Gross Motor Function Classification System (GMFCS), and the negative health outcomes. GMFCS were classified into five categories based on the ability to move: I. Walks without limitations; II. Walks with limitations; III. Walks using a hand-held mobility device; IV. Self-mobility with limitations, may use powered mobility; and V. Transported in a manual wheelchair. Since tube feeding may affect growth, GMFCS level V was then further divided into two groups: children who fed orally without a feeding tube (GMFCS V-NT) and those who had a feeding tube (GMFCS V-TF). The results indicated children with weight-for-age below the 20th percentile, GMFCS I–IV and V-NT groups had more major medical conditions than children who had weight-for-age in the 20th to 80th percentile. In contrast, the GMFCS V-TF children who had weight-for-age below the 20th percentile had less major medical conditions. Mortality rates in GMFCS level III–V were significantly higher in children with weight-for-age below the 20th percentile compared with children in the 20th to 80th percentile. In conclusion, cerebral palsy children who have very low weights have more major medical conditions and higher mortality rates. The new growth chart will help identify the risks in nutritional status and health outcomes. Brooks growth charts can be used to decide if a child with cerebral palsy needs a gastrostomy tube. For children with gastrostomy tubes, it can be used to suggest an ‘optimal’ weight.

### 4.3. Down Syndrome

Down syndrome is a common chromosomal disorder. Children with Down syndrome often have lower birth weights and slower growth rate compared to their peers due to potential issues that can affect growth. In 1988, Cronk et al. [53] conducted a study in 400 males and 300 females (1 month through 18 years) with Down syndrome, including children with congenital heart disease. However, it was recommended that previously used Down syndrome growth charts should not be used because it is no longer reflecting the current population styles and body proportion [54]. The Down Syndrome Growing Up Study (DSGS) developed growth charts using measurements from 637 Down syndrome participants, to assess growth and nutritional status [55]. Down Syndrome specific growth charts for height, weight, and head circumference are useful to monitor growth in these children from birth to 20 years, however, for children 10 years or older, the CDC BMI growth chart is a better indicator of excess adiposity [56].

## 5. Utilization of Z-Score

A Z-score describes how far a measurement deviates from the population mean while a percentile defines the position of a child in the reference population. Both percentiles and Z-score provide useful assessment in examining children’s growth. Although percentile is more straightforward and Z-score can be hard to explain to the parents, Z-score has advantages in estimating the deviation from the mean and tracking changes. When plotted on the WHO weight-for-age growth chart, the Z-score for a 5-month-old girl who weighs 4.5 kg is −3.5. The weight of this girl is 3.5 standard deviation below the average of healthy children of the same age, which is considered severely underweight. Medical and nutritional evaluation should be conducted to identify the root causes along with applying appropriate intervention to ensure the child’s proper growth. The Z-score of −1, −2, and −3 roughly corresponds to 15th, 3rd, and 1st percentile while 0, 1, 2, and 3 equivalates to 50th, 85th, 97th, and 99th percentile. The percentile is not as wide as the Z-score because the 3rd percentile corresponds to a Z-score of −2 and 97th percentile corresponds to 2. As mentioned earlier, the extremely high or low percentiles in the CDC and WHO growth charts might not be precise. Therefore, Z-score describes patients’ anthropometric status more precisely, especially when they do not fall within the growth chart percentiles. In addition, Z-score is a standardized measure and can be used to compare across different measures. WHO recommends using Z-score-based growth curves in clinical application [57]. Great efforts would be needed to use Z-score widely in public.

## 6. Resources

WHO offers the software WHO Anthro (version 3.2.2) which includes three modules: anthropometric calculator, individual assessment, and nutritional survey. CDC provides a SAS program for users to calculate percentiles, and Z-scores based on CDC growth charts. WHO releases programs in a variety of statistical software for WHO growth charts based on Z-score calculations. The CDC website: http://cdc.gov/growthcharts (accessed on 1 November 2025) is a helpful resource for detailed information in growth charts. In addition to the statistical programs provided by the CDC and WHO, newer tools that help parents and health providers identify developmental issues have become available. Digital growth monitoring applications such as CDC’s Milestone Tracker app, Child Growth Monitor app, and GrowthMonitor use the smartphone to track a child’s growth and can be monitored at home. Popular growth chart calculators such PediTools, CDC Child & Teen Calculator, Baylor College of Medicine BMI Z-score calculator and mobile apps have changed clinical practice in facilitating early diagnosis and ongoing treatment assessment. An artificial intelligence (AI)-assisted tool was developed for automating calculations of Z-scores for pediatric patients with achondroplasia [58].

## 7. Conclusions

For general populations, WHO and CDC provide growth charts graphs for health professionals or parents to plot individual child’s measurements in the graph and estimate the percentile or Z-score. This represents a convenient way to assess and track children’s growth over time. Although BMI percentiles are a reliable surrogate for adiposity, defining more accurate, age-specific BMI guidelines for children is crucial to correctly identify those at risk for obesity-related health issues. The association between long-term health risk and particular BMI values in children needs to be further investigated. Over the years, alternative growth charts have been developed for subpopulations such as premature infants, Down syndrome patients, and other conditions. These should be used in conjunction with WHO or CDC growth charts to ensure correct assessment and appropriate medical care.

## Figures and Tables

**Table 1:** Comparison of CDC and WHO growth charts.

	CDC	WHO
Type of data	Cross-sectional data	Longitudinal and cross-sectional data
Data source	One country (USA), 1963–94	MGRS in 6 different countries, 1997–2003
Sample population	National vital statistics, Missouri and Wisconsin vital statistics, starting at 2 mos of age: NHES II (1963–64), NHES III (1966–70), NHANES I (1971–74), Fels Research Institute (1929–75), NHANES II (1976–80) and NHANES III (1988–94)	Longitudinal: Children < 2 years—882 children at frequent follow-up points with 19,900 records. Cross-sectional sample: 6697 children with 8306 records (some children in Brazil and the USA had multiple measurements)
Inclusion criteria	All children	Healthy children only with optimal environmental and health conditions
Exclusion criteria	Low birth weight (<1500 g)	Children with chronic disease or sub-standard living conditions were excluded
Breastfeeding	~50% ever breastfed; ~33% breastfed at 3 mos	100% ever breastfed; 100% breastfed at 12 mos (<24 mos); 100% breastfeeding at 3 mos (24–59 mos)
Curve construction	Modified LMS method	BCPE method with curve smoothing by cubic splines
Social Determinants of Health	No exclusions based on the following: Socioeconomic status Maternal health Environmental factors	Minimize the influence of adverse conditions: Low socioeconomic status Poor maternal health Poor environmental factors Excluded: Maternal smoking during pregnancy or lactation
Clinical interpretation	Descriptive references Actual child growth patterns in the U.S. Growth relative to other U.S. children Tracking typical growth Deviation suggests nutrition issues or underlying medical conditions	Prescriptive standards Optimal growth trajectory Growth under ideal conditions Infant feeding adequacy assessment Deviation indicates potential issues associated with suboptimal nutrition, underlying health conditions, environmental or socioeconomic factors
Limitations	Data pooled from different resources Infants not excluded if not exclusive breastfeeding, chronic disease, sub-standard living condition Weight or length data birth to 2 month—not available Birth data from only two states (Wisconsin and Missouri) Percentile <3rd or >97th are not reliable/accurate May underestimate the rapid early growth rates Do not tailor to specific countries and ethnic groups	Longitudinal data only within the first 2 years; then cross-sectional Exclude children with extreme values Sample size is smaller for samples > 2 years Intended to reflect optimal growth of infants and children Do not tailor to specific countries and ethnic groups
Recommendation	Children 2–19 years	Children < 2 years of age

BCPE: Box-Cox power exponential; MGRS: Multicentre Growth Reference Study; NHES: National Health Examination Survey; NHANES: National Health and Nutrition Examination Survey.

## Data Availability

This is a review article. The research did not produce any original data.
